# News and Items

**Published:** 1867

**Authors:** 


### NEWS & ITEMS.

Dr. Horatio R. Storer the well known and distinguished teacher of Gynæcology encloses to the Journal the following notice, which we insert with great pleasure. Dr. Storer was with Simpson, of Edinburgh, as his *assistant in practice* during 1854-5, and was selected by him, as one of the editors of his Obstetric works, and both whilst abroad and since his return home has devoted his sure powers of mind and great practical genius to the developement of this important department of practice. It is unnecessary to more than merely announce the time of such a course of instruction from him to secure a large attendance.

To Physicians.—At the request of several members of the profession, Dr. Horatio R. Storer will deliver a private course of twelve lectures upon the Treatment of the Surgical Diseases of Women, during the first fortnight of June, at his rooms in Boston. Gentlemen attending the course will be required to show their diplomas. Fee $50.

*Hotel Pelham, Boston, 29th March, 1867.*

*Resignation of Prof. Gunn at the University of Michigan—Action of the Regents.*—The JOURNAL chronicles with pleasure a deserved tribute to Prof. GUNN on occasion of his leaving the University, being in the shape of a resolution adopted unanimously in full Board, April 9th, 1867:—

*Resolved*, That while we reluctantly accept the resignation of Professor Moses Gunn, M.D., who, first as Professor of Anatomy for two years, next as Professor of Surgery for thirteen years, has devoted his eminent ability and energy to the Department of Medicine and Surgery of this University from 1848, the year of its foundation, to the present time, and to whose skill as a surgeon and eloquence as a professor the department is so greatly indebted, we desire to express our high appreciation of his eminent services, and our strongest wishes for similar and, if possible, superior success in his future labors, wherever he may be.

The Board at the same meeting passed a complimentary resolution with regard to Prof. SAMUEL G. ANNOR who retires from his connection with the University to take a chair in the Brooklyn College, N. Y.

*Complimentary Letter to Dr. Lewitt.*—As an evidence of their high appreciation of his services, the Medical Class at Ann Arbor adopted and presented to Dr. LEWITT the following letter:

ANN ARBOR, March 22, '67.

*To William Lewitt, M.D., Demonstrator of Anatomy, Medical Department, University of Michigan.*

DEAR SIR: Knowing, as we well do, the difficulties attending the position you occupy, as Demonstrator of Anatomy, the energy necessary to supply so large a class with *materiel*—none of which having been furnished by this State—allow us to congratulate you on your success; and, as members of the Class of 1866-'67, to express our entire satisfaction with the manner in which your department has been conducted.

For while other medical schools during the past winter have felt most sorely what it is to be in *want*, we have had the benefit of a plentiful supply of the *best*, with an abundance to spare. And when it is remembered that the Class you have assisted so efficiently during the past session, in acquiring a knowledge of that most important branch of medicine—practical anatomy—is the largest that ever assembled in America, or perhaps in the world, too much praise cannot be bestowed upon our "little Napoleon of the dissecting room."

In conclusion, we beg to tender you our sincere thanks, and best wishes for your future welfare and prosperity.

PROF. M. GUNN, of the Chair of Surgery in Rush Medical College, has now removed to this city, and may be found or addressed at his office in the "McCormick Building," Southeast corner of Randolph and Dearborn streets. Dr. G. has purchased for his residence that beautiful property on the lake shore, corner of Calumet Avenue and Twenty-First street. He will, it is understood, confine his practice exclusively to Surgery.

Dr. LEWITT, the Demonstrator of the College, will be found at the same office, and engages both in Surgery and general practice.

*Southern Journal of Medical Sciences*—Edited and published by D. Warren Brickell, M.D., C. Read, M.D., N. S. Mitchell, M.D., and Joseph Holt, M.D., commences its second volume May 1st, 1867. Two hundred pages are issued quarterly in a style which compares favorably with *Am. Jour. Med. Sci.* and other older quarterlies. It is evidently prepared with great industry, and is edited with signal ability. We commend it strongly to such of our readers as desire to peruse a high toned and valuable organ of the profession. $6.00 *per annum.* Communications to be addressed Lock Box 890, New Orleans P. O.

*New Medical Journal.*—C. A. LOGAN, M.D. and T. SINKS, M. D., of Leavenworth, Kansas, have associated in the editing and publication of a new monthly to be entitled "*The Leavenworth Medical Herald.*" Its subscription price is placed at $3. From what we know of the editors we believe it will herald true medical progress, and will be (forgive it Bro. Logan,) *leaven worth* trying for professional bread. Here is our ☞.

*University of Michigan.*—The chair of surgery in the University of Michigan has been filled by the appointment of WARREN GREEN, M.D., an alumnus of the college. The chair of Homœopathy is yet vacant, although it is rumored the Regents propose to appoint to it the present professor of Chemistry, S. H. DOUGLAS, M.D., who is said to be an applicant.

*Medical Bookstore.*—A recent conflagration in the upper stories of the building occupied by W. B. KEEN & Co., the favorite medical booksellers of this city, as an incident to the means adopted to extinguish it, dampened a large proportion of their then stock on hand, but not a particle of their energies. The telegraph and express have already fully replenished their stock, and correspondents may feel sure of being supplied at once with all the standard and recent medical works at as low a price as in any eastern city, thus saving both time and money. Our readers will find them always prompt, reliable, and "on the square."

*Meeting of the Societies.*—Attention is called to the notices of the ensuing meetings of the American Medical Association and of the Illinois State Medical Society, in the present number of the JOURNAL. The present condition of the profession in this State, and throughout the country, is such as to render attendance almost imperative upon them. Rich returns may be expected from the careful observations made during the war, not only upon surgical and general practice, but especially upon hygiene. Just now, and we fervently trust it will continue so during all coming time, this latter awakens vastly more attention, and incites to profounder study, than mere therapeutics.

*International Medical Congress at Paris.*—The International Medical Congress will be opened at Paris on the 16th of August next. The Central Committee earnestly desire an active participation in the Congress on the part of Medical

Societies from all parts of the world, by sending delegates to represent them.

By the third article of the statutes, foreign delegates are admitted without any pecuniary consideration.

The undersigned having been appointed a Corresponding Delegate by the Central Committee at Paris, would urge upon Medical Societies the propriety of appointing delegates to the Congress as speedily as practicable, and reporting them to him, that he may forward them as early as possible to the Central Committee.

S. W. BUTLER, Philadelpha, Pa.
*Corresponding Delegate.*

*The International Medical Congress.*—The following note from Prof. Miller explaines itself. It is understood that Prof. Freer has already secured his stateroom in a European steamer for the 6th of June.

RUSH MEDICAL COLLEGE, Chicago, April 2, 1867.

EDITOR CHICAGO MEDICAL JOURNAL:—At a regular meeting of the Faculty of Rush Medical College, held this day, Prof. Joseph W. Freer, M.D., was duly elected to represent said College in the International Medical Congress, which is to assemble in the city of Paris the 15th of August next.

DE LASKIE MILLER, Sec'y.

*Illinois State Medical Society.*—The next annual meeting of this society will be held in the hall of the House of Representatives, at Springfield, Ill., commencing on the *first Tuesday in June*, at 10 o'clock A.M. We are authorized by the Committee of Arrangements to state, that the Chicago, Alton and St. Louis, the Toledo, Wabash and Great Western and the Illinois Central railroads will pass members of the Society to and from the meeting, over those roads for *one fare and one-fifth.*

*Editor Chicago Medical Journal:*—The STATE MEDICAL SOCIETY OF INDIANA will convene at Indianapolis on, *Tuesday*, May 21st, 1867.

Very Respectfully, L. D. WATERMAN, *Secretary.*

COMMITTEE ON PRACTICAL MEDICINE AND EPIDEMICS.

Physicians, throughout the State, are earnestly requested to forward to either of the members of the committee, all communications, on this department, on or before the 15th day of May inst.

*Committee.*
- J. ADAMS ALLEN, M.D., box 1948, Chairman Chicago.
- L. T. HEWINS, M.D., Loda, Iroquois county.
- R. E. McVEY, M.D., Waverly, Morgan county.

*Erratum.*—By an oversight of the proof reader of the March number of the JOURNAL, the case reported by G. B. Lester, M.D., (p. 112) in the third line of the third paragraph should, but does not, commence and read as follows:

"The edges of the wound were everted, and blood was oozing from *the* whole cut surface, lighter colored than natural and frothy; *hand* slightly swollen and painful."

The italicised words indicate the needed corrections.

*Married.*—At the residence of Geo. W. Moody, Esq., White River, Mich., by the Rev. L. J. Griffin, Lucian D. Clarke, M.D., and Miss Anna W. Young, both of Chicago.

*Obituary.*—Howard Townshend, M.D., died at Albany, N.Y., on the 16th of January, 1867. At the time of his death, Dr. Townshend was a professor in the Albany Medical College, and an influential member of several official boards for which his scholarly acquirements and large experience eminently fitted him. His death may well be considered a public loss.

*Trimmed or Untrimmed.*—Subscribers generally prefer to have the leaves of the JOURNAL uncut, so that when bound in a volume it may have a wider margin. But all do not agree as to the æsthetics of the matter, of which discrepancy let the following note, inserted *verbatim et literatim et punctuatim*, be for a testimonial: (Names omitted.)

"Dear sir—you will pleas stop My subscription for the Journal this is the second Notes i have gave you or at least the second letter i have sent you I can get as good a Journal all ready Trimed for 2 per year Yours"

As both the first and second "*Notes*" were sent to the former editors, "our withers are unwrung." Meanwhile we cogitate which most needs to be "*Trimed*" the *Journal's* edges or the "preliminary education" of our predecessor's correspondent.

*Back Numbers of the Journal Wanted.*—JULY and AUGUST, 1865, and JANUARY 1866. If any of our subscribers happen to have spare copies of these numbers of the JOURNAL, they will confer a favor upon several subscribers who need them to complete their sets, by enclosing them to the Editor, Box 1948, who will be happy to reciprocate in any manner suggested.

---

ADVERTISEMENT.

FINE LITHOGRAPHS of the late Professor BRAINARD, also of Professors GUNN, REA, MILLER, and ALLEN, can be obtained at Twenty-Five Cents each, by addressing or calling upon Charles Keil, Janitor Rush Medical College.

---

RECEIPTS ACKNOWLEDGED FROM:—J. J. Oakley, $2; E. M. Whipple, 2; J. L. Prentiss, 2; James Muncy, 5; J. T. Wilson, (Wisconsin Insane Hospital,) 2; D. D. T. Hamlin, 4; John Swain, 2; John G. DeWolf, 2; Edw. Thomas, 2; Jas. Prentice, 2; W. C. Brown, 2; J. W. Thayer, 2; M. G. Smith, 2; O. W. Cline, 2; N. S. Smith, 2; A. Taylor, 6; Wesley Anderson, 1; H. Symmes, 2; —— Russell, 3; A. C. Jackson, 8; Thomas Johnson, 2; D. Kirkpatrick, 2; Geo. F. Keiper, 2; P. M. Martin, 2; John Becker, 2; B. F. Ross, 2; F. R. Payne, 2; J. A. Hatch, 3; J. Low, 2; J. W. Saucerman, 2; L. D. Tompkins, 2; A. A. Rawson, 2; G. R. Bibb, 2; T. H. Parks, 2; R. W. Earll, 2; L. T. Strother, 2; W. L. Whitney, 2; S. Marks, by S. C. White, 2; J. B. Groesbeck, 2; D. E. Ellis, 2; A. A. Hoy, 8; V. L. Hurlbut, 2; J. W. Trabue, 2; P. M. McFarland, 2; L. D. Lowell, 2; A. Hard, 2; F. W. Byers, 2; J. Lamburn, 2; G. B. Lester, 2; K. E. Rich, 2; C. B. Reed, 2; E. J. Shelton, 2; O. D. Coleman, 5; H. C. Godfrey, 2; Jas. P. Tucker, 2; F. C. Robinson, 2: G. H. Tebo, 2; W. E. Schenck, 2; Jas. L. Gandy, 2; S. W. Noble, 2; David Dodge, 2; Alfred Waterman, 2; N. P. Holden, 2; A. W. Lueck, 2; W. H. Clutter, 2; C. H. Oleson, 2; R. B. Wetmore, 2; Isaac

Snyder, 2; Hosea Davis, 2; L. Carr, 2; A. Holliday, 2; Wm. H. Vance, 2; C. B. Ames, 2; E. S. Edwards, Milton, Iowa, 2; H. Steele, 10; H. B. Fawcett, 2; E. W. Edwards, 2; W. B. Hagard, 5; H. A. Guthrie, 2; Geo. B. Christie, 2; F. G. Kuener, 2; R. J. Brackenridge, 2; B. L. Ashbaugh, 2; A. G. Conner, 2; A. Hodge, 2; A. B. Younkman, 2; D. S. Waddel, 2; Chas. Knapp, Jr., 2.

---

*Mortality Report for the Month of February:—*

CAUSES OF DEATH.

| | | | |
|---|---|---|---|
| Accidents, | 6 | Fever, Congestive, | 3 |
| Asthma, | 1 | Fever, Remittent, | 2 |
| Abscess, | 2 | Fever, Scarlet, | 14 |
| Bronchitis, | 1 | Fever, Typhoid, | 10 |
| Cancer, | 3 | Hydrocephalus, | 4 |
| Cold, | 1 | Inflammation of Bowels, | 4 |
| Croup, | 8 | Inflammation of Brain, | 5 |
| Consumption, | 42 | Inflammation of Lungs, | 19 |
| Convulsions, | 18 | Inflammation of Stomach, | 1 |
| Childbed, | 5 | Inflammation not stated, | 1 |
| Congestion of Brain, | 7 | Killed, | 1 |
| Congestion of Lungs, | 2 | Marasmus, | 2 |
| Delirium Tremens, | 1 | Neuralgia, | 1 |
| Decline, | 2 | Old Age, | 6 |
| Diarrhœa, | 1 | Paralysis, | 1 |
| Diphtheria, | 6 | Pneumonia, | 1 |
| Dropsy, | 9 | Phthisis Pulmonalis, | 2 |
| Dysentery | 1 | Rheumatism, | 1 |
| Disease of Brain, | 1 | Stillborn, | 22 |
| Disease of Bowels, | 2 | Small-Pox, | 5 |
| Disease of Heart, | 4 | Teething, | 9 |
| Disease of Liver, | 2 | Whooping-Cough, | 1 |
| Disease of Lungs, | 2 | Worms, | 1 |
| Disease of Throat, | 1 | Gun-shot Wound, | 1 |
| Epilepsy, | 1 | Ulcer, | 1 |
| Erysipelas, | 2 | Unknown, | 1 |
| Total, | | | 255 |

Ages of the Deceased.—Under 5 years, 118; over 5 and under 10 years, 17; over 10 and under 20, 10; over 20 and under 30, 35; over 30 and under 40, 31; over 40 and under 50, 16; over 50 and under 60, 8; over 60 and under 70, 10; over 70 and under 80, 6; over 80 and under 90, 2; unknown, 2. Total, 255.

NATIVITIES.

| | | | | | |
|---|---|---|---|---|---|
| Chicago, | 124 | Germany, | 30 | Scotland, | 2 |
| Other States, | 39 | Ireland, | 33 | Wales, | 1 |
| Canada, | 1 | Norway, | 3 | Unknown, | 5 |
| England, | 12 | Nova Scotia, | 1 | | |
| France, | 1 | Sweden, | 3 | Total, | 255 |

DIVISIONS OF THE CITY.

| | | | | | | | |
|---|---|---|---|---|---|---|---|
| North, | 67 | South, | 81 | West, | 106 | Total, | 255 |

*Mortality Report for the Month of March:—*

CAUSES OF DEATH.

| | | | |
|---|---|---|---|
| Accidents, | 2 | Fever, Scarlet, | 10 |
| Abscess, | 1 | Fever, Typhoid, | 5 |
| Bronchitis, | 1 | Fever, Childbed, | 2 |
| Cancer, | 2 | Fever, Typhus, | 1 |
| Colds, | 6 | Hydrocephalus, | 10 |
| Croup, | 8 | Inflammation of Bowels, | 7 |
| Consumption, | 40 | Inflammation of Brain, | 3 |
| Convulsions, | 43 | Inflammation of Lungs, | 11 |
| Childbed, | 4 | Inflammation of Throat, | 1 |
| Congestion of Brain, | 1 | Inflammation, not stated, | 2 |
| Congestion of Lungs, | 7 | Killed, | 1 |
| Delirium Tremens, | 1 | Marasmus, | 1 |
| Drowned, | 1 | Measles, | 1 |
| Decline, | 1 | Old Age, | 9 |
| Diphtheria, | 5 | Poisoning, | 1 |
| Dropsy, | 7 | Paralysis, | 3 |
| Disease of Brain, | 1 | Pneumonia, | 2 |
| Disease of Bowels, | 1 | Rheumatism, | 1 |
| Disease of Heart, | 4 | Suicide, | 1 |
| Disease of Liver, | 2 | Stillborn, | 8 |
| Disease of Lungs, | 5 | Spasms, | 4 |
| Disease of Hip, | 1 | Small Pox, | 3 |
| Erysipelas, | 1 | Teething, | 12 |
| Fever, Congestive, | 6 | Whooping-Cough, | 10 |
| Fever, Remittent, | 3 | Ulcer, | 1 |
| Fever, Spotted, | 1 | Unknown, | 15 |

| | |
|---|---|
| Total, | 280 |
| Total number last year for the month of March, | 254 |
| Increase, | 26 |

DIVISIONS OF THE CITY.

North, 67 | South, 90 | West, 123 | Total, 280

| | |
|---|---|
| Total number during the month of March, | 280 |
| Total number during the month of February, | 255 |
| Increase, | 25 |

Ages of the Deceased.—Under 5 years, 143; over 5 and under 10 years, 16; over 10 and under 20, 16; over 20 and under 30, 29; over 30 and under 40, 27; over 40 and under 50, 17; over 50 and under 60, 7; over 60 and under 70, 9; over 70 and under 80, 10; over 80 and under 90, 2; over 100, 2; unknown, 2. Total, 280.

NATIVITIES.

